# Would Household Medical Expenditure Affect Chinese Citizens to Have More Children?

**DOI:** 10.3389/fpubh.2022.902896

**Published:** 2022-07-13

**Authors:** Jing Chang, Meixin Wang

**Affiliations:** Department of Finance and Economics, School of Economics, Qingdao University, Qingdao, China

**Keywords:** willingness of having multiple children, household medical expenditure, aging society, public health expenditure, demographic decline

## Abstract

This research investigates how household medical expenditure affects Chinese citizens having more children. We examined the causal relationship and found a negative influence of household medical expenditure on the willingness to have more children, confirming that increasing household expenditure on medical care may specifically decrease the high willingness. Chinese policymakers should adopt appropriate and effective strategies to mitigate the potential negative effects of household medical expenditure on the birthrate. These analyses emphasize the importance of promoting economic growth, increasing public health expenditure, and increasing medical security in the context of population imbalance and the trend of a rapidly aging society in order to encourage people to have more children, thereby slowing population decline.

## Introduction

The primary goal of this paper is to determine whether or not household medical expenses influence Chinese citizens' decision to have multiple children. The development of a country and the development of its population are inextricably linked. After 40 years of the national one–child policy, China's fertility rate has continued to decline, and the working–age population resumed its growth trend in 2013. The demographic dividend gradually decreases, and the pressure of population aging increases. In 2020, China's total fertility rate was only 1.3, lower than the internationally recognized value of 1.5. In July 2021, China and the Health Commission announced that the low birth population would be continued. The demographic structure was unbalanced during this period. If things continue as they are, China will most likely enter a developmental vision of population aging, declining economic vitality, and “getting old before getting rich”. In order to encourage the healthy development of the demographic structure, the Chinese government implemented the “single two–child” policy^1^ in 2013. All couples could have two children in 2015 officially. The bureau developed policies to implement the policy that “a couple can have three children.” In recent years, the population policy has changed from “only child” to “three children.” The policy revolution demonstrates the Chinese government's determination to change population structure and address aging issues.

The actual population growth is generally lower than expected on a national scale. In 2016, the birth rate was 13.57% and the country's new population was 17.86 million, significantly increasing from the 16.55 million born in 2015 (the birth rate was 11.99%. Furthermore, the country's new population was 17.23 million in 2017(the birth rate was 12.67%), a slight decrease from 2016. The new population's birth rate in 2018 was only 10.94%, the lowest value in history since China's establishment. We intend to look into the reason for this. The willingness to bear children (1) refers to people's intuitive feelings about fertility behavior, mentioning the desire to bear children without any interfering factors, such as the number of children, gender, and the time of childbirth. The fertility rate is largely affected by the willingness to bear children, and economic factors may influence the desire (2). Data from the 7th China Census show that household medical expenditures have increased significantly as China's population ages. According to statistics, medical and health expenditures have the highest growth rate among national per capita consumption expenditures in 2019. By 2020, the average annual per capita medical and health expenditures of Chinese residents have increased to 1,843 yuan, accounting for 8.65% of annual total household consumption expenditures. The increase in family medical expenditure has further increased the economic burden on residents. Do household medical expenses influence Chinese residents' willingness to have more children?

Based on this, this article considers family medical care as a proxy variable of the family economic burden, using the 2017 Chinese General Social Survey's (CGSS) data to build a binary discrete selection model to study whether family medical expenditures of Chinese residents have an impact on residents' willingness of fertility under the background of Chinese fertility revolution policy. Investigating the causes, avoiding social risks, and promoting related research in healthy population development are the priorities.

## Literature Review

The economy is a crucial factor affecting the willingness to give birth, including macroeconomic and microeconomic factors. On a macro-level, if the economy is doing well and job opportunities are plentiful, the likelihood of early marriage and willingness to have children will increase. A recession and rising unemployment, on the other hand, will decrease people's desire to marry and have children (3). Economic difficulties will also encourage the development of large-scale birth control policies (4). This law has been proven by several economic downturns in European and American countries (5). Economic modernization is also the process of shifting economic production away from agriculture and toward industry. The economic production of the agricultural society emphasizes the power of many people, and people have an innate desire to have more children. However, the economic production of the industrial society emphasizes the maximization of profit, labor time, and production efficiency, and it “essentially does not encourage fertility.” Generally speaking, the higher the female labor force participation rate, the lower the fertility rate (6). On a micro level, household savings and disposable income significantly impact willingness to bear children (7). Dual-employee families have more financial resources and are more willing to have children. Unemployed families, particularly male unemployment, generally suppress their willingness to reproduce. Nevertheless, female unemployment will have a substitution effect-more time giving birth and supporting children. It increases the couple's willingness to have children (8), but under normal circumstances, mothers' loss of income remains one of the primary reasons for restraining fertility (4). Thus, fertility has declined, siblings, aunts, and paternal relationships have gradually faded, resulting in an overall shrink in family size. Reduced transfer of funds within the family and funds for mutual support will further reduce willingness to bear children (9), and vice versa. Furthermore, there is a positive correlation between family credit opportunities and fertility (10).

Social security has a redistributive effect and is closely related to childbearing willingness. However, the impact could be bidirectional. On the one hand, the “substitution effect,” which serves as a “prevention of old age,” can reduce the willingness to bear children (11). An increase in the level of social security can alleviate the pressures of old age while also lowering the fertility rate (12). In contrast, it is the “subsidies effect,” social security may increase willingness to give birth by providing a certain amount of relaxed fertility time to compensate for the opportunity cost of fertility. Nonetheless, it has no discernible effect on high-income families (13). Furthermore, the elderly can only obtain lower payments via social security redistribution (14). The willingness to bear children is heavily influenced by social pressure and social capital (15). Furthermore, medical insurance has a variable effect on the willingness to have a second child. Subsidized social security can help increase the desire to have children, whereas an excessively high social security payment burden has a restraining effect (16).

Previous research on the willingness to bear children included social security research, but it was limited to examining the impact of “whether to participate in these guarantees (including endowment insurance or medical insurance)” on the willingness to bear children. Few studies on the effect of family social security expenditure levels on the willingness to bear children have been conducted at the microscopic level. China formally implemented the “three–child” birth policy on June 1, 2021. The majority of domestic research in China focuses on the willingness to have two children. Based on the most recent population policy reform, we examine the medical burden of Chinese families from a micro perspective. The ratio of family medical expenditure to family income is used as a proxy variable for the family's economic burden in order to investigate its influence on Chinese families' willingness to organize childbirth. Based on previous experience, this article divides other control factors into individual factors and social factors using CGSS data.

## Introduction of Chinese Fertility Revolution

**Figure 1:**
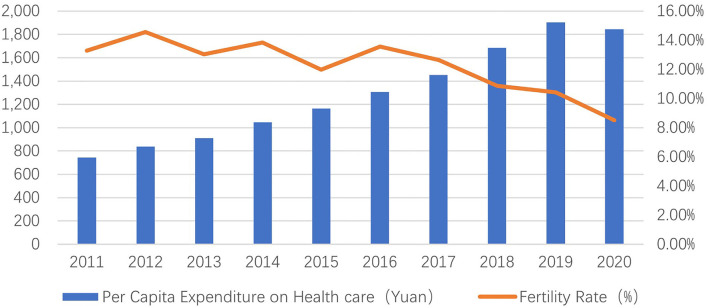
The fertility rate in each year (2011–2020).

At the beginning of China's establishment, the population economy grew rapidly to expand the population brought about by the war. The government encouraged the residents to give birth by implementing policies, and the population growth increased. The growth rate and the national family planning policy were incorporated in 1978, which became the basic national policy at the 12th National Congress of the Party. The birth rate was effectively controlled and continued in 1990. Since 2010, the recovery of production, insufficient living, and low birth population have been serious in China. The “Separate Two” policy was adopted at the Third Plenary Session of the 18th National Congress of the Communist Party of China in 2013 to promote the healthy development of the population and maintain stability. It was widely implemented nationwide at the end of 2014. However, after the policy was made public, the birth rate did not rise as expected, and the population is still growing rapidly. The level is low: between 2013 and 2015, the birth rates were 12.08, 12.3, and 12.07%. The “Comprehensive Two-Child Policy” was passed at the Fifth Plenary Session of the 18th National Congress of the Communist Party of China in 2015 to effectively increase the annual birth rate of the population and improve the production problem, implying that one couple of parents can have two children. Our country's birth rate has increased since the implementation of this policy. In 2016 and 2017, the birth rate was 12.95 and 12.43%, but in 2018 and 2019, the birth rate decreased again to 10.94 and 10.48%, respectively. As shown in Figure 1, the birth rate of China's population has increased significantly after implementing the two birth policies, but it began to decline sharply after 2016, and it is still urgent to solve the problem of a low birth rate. China officially launched the “three-child” policy in the first half of 2021 to encourage couples to have three children.

## Data Source and Model Construction

### Data Source

The data for this study came from the 2017 Chinese General Social Survey (CGSS) questionnaire. CGSS, which began in 2003, is China's first national, comprehensive, and ongoing academic survey project. It collects data at multiple levels of society, communities, families, and individuals, covering 125 counties (districts) in the United States and 500 in China. There are sub-districts (townships), 1,000 neighborhood (village) committees, and 10,000 individual households. The CGSS2017 survey data has a sample size of 12,583 people. This article studies the willingness to bear children and its influencing factors, combined with the marriage law. The population between 20 and 49 years is selected as the research sample in the present studies. The variables related to the willingness to bear children are determined, and invalid data is deleted (After the sample's variable responses are missing, obviously unreasonable, etc.), 5,361 valid samples are eventually retained.

### Model Construction

Because the willingness of fertility is a non-continue variable, we used the Logistic model to analyze. The regression model is as follow:


$$\begin{array}{l} p_{i}=\frac{\exp(Zi)}{1+\exp(Zi)},\;pi=E(will=1|{\text{medical}}_{\text{i}},{\text{personal}}_{\text{i}},{\text{social}}_{\text{i}})\\ \;Zi=\partial 0+\partial 1{\text{medical}}_{\text{i}}+\;{\text{personal}}_{i}+\;{\text{social}}_{i}+\;\mu_{i} \end{array}$$

Dependent variable: we put the willingness to bear more children (will) as the dependent variable. In the CGSS survey questionnaire, there is a question that asked, “If there are no policy restrictions, how many children do you want to have?” The sample with an answer of 0 or 1 is set as “0”, which means the respondents are unwilling to have more children; and the sample with an answer > 1 is set to “1”, which means the respondents are willing to have more children.

Independent variable: The major independent variable of this article is the level of family medical expenditure. In the CGSS survey questionnaire, there is a question that asked, “In the total expenditure of your family last year, what is the medical expenditure (excluding the amount of medical insurance reimbursement)?” We started by calculating the amount of family medical expenses in total income. The ratio was then divided into five levels, with 3 representing the average level; 1 and 2 representing significantly higher or higher than the average level, respectively; 4 and 5 representing significantly lower or far lower than the average level, respectively. As the number grows, the level of family medical expenditure decreases.

We put other variables into two parts of basic personal characteristics (personal) and social characteristics (social).

Among them, the basic personal characteristics of individuals include the respondent's gender, age, ethnicity, physical health, education, marital status, and the number of people living together in the family. Social characteristics include whether they believe in religion, whether they participate in social security projects, and the location of their household registration.

Table 1 presents the description and assignment of the independent variables.

**Table 1:** Description of independent variables.

**Variable**		**Questions**	**Description**
Level of family medical expenditure		How much of family medical expenditure? What is the total income for the whole year?	Take “1” if much larger than average, Take “2” if larger than average, Take “3” if same as average, Take “4” if lower than average, Take “5” if much lower than average
Basic Individual characteristics	Sex	Sex	Male take “1”, Female take “0”
	Age	What is the year you were born?	2017-year you born
	Nation	nation	Han take “1”, others take “0”
	Health	What do you think about your health?	Take “1” if very unhealthy Take “2” if not healthy, Take “3” if average level, Take “4” if healthy, Take “5” if very healthy
	Education level	What is your highest education level	Unfinish primary school take “0”, finish primary school take “6”, finish the junior middle school take “9”, finish the senior high school or technical secondary school take “12”, finish the junior college take “15”, obtain an undergraduate degree take “16”, get a graduate degree take “19”
	Marital status	What is your marital status?	“not married”, “live together”, “widowed” take “0”, “first marriage”, “second marriage” take “1”
	Family members	How many are your family members?	Take numbers
Social characteristic	Religion	Do you have religious beliefs?	Yes as “1”, No as “0”
	Social insurance	Do you participate in social insurance program?	Yes as “1”, No as “0”
	Resident	Household registration type	Agricultural registered residence &Blue print account take “0”,
			Non-agricultural registered permanent residents take “1”

Table 2 depicts the descriptive statistical analysis of the assigned variables and the results.

**Table 2:** Variables statistics.

	***N***	**Max**.	**Average**	**Standard**
				**error**
Willingness of fertility	5,361	1	0.7461	0.43527
Level of family medical expenditure	5,361	5		
Sex	5,361	1	0.4579	0.49827
Age	5,361	49	36.335	8.479
Nation	5,361	1	0.92	0.271
Level of healthy	5,361	5	3.88	0.949
Education level	5,361	19	11.0657	4.29315
Marital status	5,361	1	0.7678	0.42230
Family economic level	5,361	5	2.63	0.711
Family members	5,361	16	3.12	1.416
Religions	5,361	1	0.0899	0.28608
Social insurance	5,361	1	0.9355	0.24574
Registered permanent residence	5,361	1	0.4574	0.49823

The mean value of the dependent variable's willingness to have more children is 0.7462, implying that the sample who wants to have two or more children accounts for 74.62% of the total number of samples. As seen from the analysis, most respondents want to have more children.

The sample's gender, age, ethnicity, and religious beliefs are classified. The sample's multiple choices willingness to be classified are descriptive gender statistics. As shown in Table 3, the classification is based on gender statistics, and it is found that the difference between males and females is not big. The longer they age, the older, the younger, the younger, the younger, the younger, the older, the younger, The older the residents are, the more inclined they are to have more children, and the fertility intention of the residents in their 40 s is nearly 9 percentage points higher than that in their 20 s. According to the classification, rural residents with a large number of children are willing to choose smaller urban residents and more than ten grains. Furthermore, we also know that the will of the non-Han people is higher than that of the Han people, and the fertility intension of religious residents is higher than that of non-religious residents.

**Table 3:** Descriptive statistics of the willingness to give birth to more children by classifying the sample.

**Independent Variable**	**The willingness to give birth to more children**	
	**Yes**	**No**
Male	0.7381	0.2619
Female	0.7529	0.2471
Age 20–29	0.7047	0.2953
Age 30–39	0.7232	0.2768
Age 40–49	0.7901	0.2099
City	0.6876	0.3124
Village	0.7955	0.2045
Han nation	0.7411	0.2589
Non-han nation	0.8037	0.1963
Have religion	0.8465	0.1535
No religion	0.7362	0.2638

## Empirical Analysis

### Basic Regression

When categorical variables are chosen as dependent variables in econometrics, the logistic regression model is typically used for analysis. The willingness to have multiple children is defined as the dependent variable in this paper, and the numbers “1” and “0” represent a willingness to have multiple children and an unwillingness to have multiple children, respectively. As a result, a binary logistic regression model was used to study and analyze the data. Models 1 and 2 gradually added variables from the two dimensions of basic personal characteristics and social characteristics while introducing the core variable of family medical expenditure. Because the model based on cross–sectional data is prone to heteroscedasticity issues with random error terms, serious heteroscedasticity issues would affect the results of model estimation and model testing. Based on this, this study first examined white heteroscedasticity in the two models mentioned above. Table 4 presents the results of the analysis.

**Table 4:** White heteroscedasticity results.

	**χ^2^**	***p***
Model 1	262.667	0.000
Model 2	341.897	0.000

Table 4 shows that the *p*-values of the two models are both < 0.05, rejecting the null hypothesis of no heteroscedasticity, indicating that the models have heteroscedasticity issues; thus, the Robust standard error regression method is used for analysis and research.

Table 5 depicts the results of regression analysis.

**Table 5:** Robust standard error regression method.

	**Model 1**	**Model 2**
Level of family medical expenditure	0.028** (3.170)	0.027** (3.079)
Sex	−0.000 (−0.040)	−0.002 (−0.152)
Age	0.001 (1.731)	0.003** (2.920)
Healthy	0.004 (0.600)	0.005 (0.725)
Han nation	−0.049* (−2.432)	−0.028 (−1.367)
Education	−0.008** (−5.363)	−0.002 (−1.206)
Marital	0.048** (2.652)	0.043* (2.385)
Family members	0.031** (7.542)	0.028** (6.874)
Religion		0.086** (4.644)
Social insurance		0.050 (1.941)
Registered residence		−0.089** (−6.269)
Constant value	0.606** (11.409)	0.479** (8.285)
*R* ^2^	0.030	0.041
F–value	*F* (8, 5,352) = 22.833**	*F* (11, 5,349) = 22.778**

* *p < 0.05*;

** *p < 0.01 in the brackets are t-value*.

Table 5 shows that the coefficient of family medical expenditure is positive, indicating that the lower the level of family medical expenditure, the more people tend to have more children. This is because a low proportion of family medical expenditure in total income indicates that the family's financial burden is relatively light, thereby more births and financial ability to raise children.

As a result, we came to the following results:

#### Basic Characteristics of the Individual

(1) The regression coefficient of age is significantly positive, indicating that residents' willingness to have multiple children will increase with age. The sample was divided into three age groups to study the differences in willingness to bear children among various age groups: 20–29 years old, 30–39 years old, and 40–49 years old were chosen as the reference groups with two dummy variables included. Repeat the regression analysis, and the results are shown in Table 6.

**Table 6:** Robust standard error regression method for different age level.

	**Model 1**	**Model 2**
Age	−0.028 (−1.534)	−0.052** (−2.793)
20–29		
Age	−0.065** (−4.607)	−0.072** (−5.109)
30–39		
Constant value	0.668** (16.774)	0.591** (12.933)
*R* ^2^	0.034	0.044
F–value	*F* (9, 5,351) = 21.972**	*F* (12, 5,348) = 22.240**

* *p < 0.05*;

** *p < 0.01 in the brackets are t-value. Because of the length of the article, only the regression results of dummy variables are retained, and the others are omitted*.

Model 2 has negative and significant regression coefficients for the 20–29 age group; both models have negative and significant regression coefficients for the 30–39 age group. It reveals that respondents under the age of 40 are less willing to have more children than those aged 40 to 49. As a result, residents aged 40–49 are more inclined to have more children than residents of other ages. There could be two reasons for this: one is that residents of this age group have already given birth to children, so they have a stronger desire to pursue the birth; the other is that they are relatively older and have more property accumulated, and they face the difficulties associated with having children. People in other age groups have less economic pressure than those in their forties and fifties. And the majority of the 20–39-year-old respondents are in the early stages of their careers. Having multiple children requires a significant amount of time and energy, which may impede their career development and increase, resulting in a decrease in their willingness to bear children.

(2) In both models, the regression coefficients for the variable ethnic group are all negative, indicating that ethnic minority compatriots are more willing to have multiple children, related to ethnic minority population policy. Because most ethnic minorities live in remote areas of our country, the area is large, sparse, and extremely small population density. When the family planning policy is implemented in remote areas, the childbirth policy of ethnic minorities is “In ethnic groups with a population of <10 million, two children are allowed, some can have three children, and four children are forbidden. (17)” As a result, ethnic minority residents have a proclivity for having multiple children.

(3) The regression coefficients of education level are all negative in both models, indicating that education level is inversely proportional to the willingness to have more children. The higher the educational background of the residents participating in the survey, the less willing they are to have more children. The reason for this could be that highly educated residents place a greater emphasis on developing their children's abilities in all areas. During their children's infancy, they should receive early education. In addition to receiving basic curriculum guidance at school, they must also pursue their interests and hobbies. As a result, the economic costs of raising children are higher, the time costs necessitate more energy, and the desire to have more children is decreasing. It is also possible that the residents' high education level implies that they have higher incomes and better work units because they rely on themselves. Savings and an old–age pension are sufficient to provide for the elderly, and there is no longer a need to rely on the traditional “child–raising for old age” method.” As a result, when compared to residents with low education levels, they are less likely to have more children. Finally, the higher the level of education, the longer it takes to have a child, which impacts the number of children born (18).

(4) Married people have a stronger sense of family responsibility than unmarried people (19), and they can be more responsible for their children, making them more likely to have more children. Furthermore, the marriage certificate provides legal protection for both spouses and their children, and married people may have only one child. In contrast, unmarried people may only plan to have one child because they have not yet given birth. For them, there are no plans to have more children. Hence, plan accordingly so that married people want children more than unmarried people.

(5) The willingness to have more children is directly proportional to the number of residents living together in the family. The number of people living in a family generally represents the number of people living with their parents and relatives (17). As a result, the more people who live in a family, the more people who can help take care of the children, and the less pressure on raising children to mature, increasing residents' willingness to have multiple children (9). Most families pursue “same family for several generations” under the influence of traditional Chinese virtues such as “respecting the old and loving the young, keeping the young in order” and other excellent virtues. It matters whether you want to have more children.

The regression coefficient values of gender and physical health status are not significant.

#### Social Characteristics

(1) The regression coefficient of religion is significantly positive, implying that religious believers are more willing to have multiple children. Respect for life and encouraging fertility is the concept of fertility held by most religions. For example, Buddhism believes that all living beings are equal and advocates “the reproduction of species”; religions such as Judaism, Catholicism, and Islam oppose abortion, contraception, and birth control; Taoism's “sects are valuable for life” and emphasize fertility (9), all of which will lead to the increase of residents' willing to bear children.

(2) The regression coefficient of the place of household registration is < zero, indicating that rural residents are more likely to have more children. The traditional concept of “raising children to prevent the elderly” is stronger in rural areas. However, most rural families are engaged in agricultural production activities, needing more labor. Thus, the cost of childcare in rural areas is lower, increasing the number of children.

(3) The regression coefficient of social insurance is not significant. It may be because the substitution and the subsidy effects cancel each other.

### Differential Influences Exist in the Intentions of Urban and Rural Residents

We grouped and regressed the samples based on the location of household registration to investigate the differential impact of family medical expenditures on the willingness of Chinese rural residents and urban residents. We found that the impact of family medical expenditures on the desire to give birth remained stable. The lower the family medical expenditure, the greater the likelihood of having more children. The overall factors of family medical expenditure have no difference in their impact on the willingness of urban and rural residents to bear children, which is consistent with the results obtained from the regression model. However, individual factors such as physical health and marital status have different effects on the willingness of urban and rural residents to have multiple children, as shown in Table 7.

**Table 7:** Sub–sample regression analysis result.

	**Village**	**City**
Family medical expenditure	0.025* (2.225)	0.027* (1.980)
Sex	−0.013 (−0.824)	0.014 (0.765)
Age 20–29	−0.067** (−2.789)	−0.030 (−1.011)
Age 30–39	−0.058** (−3.239)	−0.088** (−3.991)
Healthy	−0.005 (−0.699)	0.023* (2.063)
Nation	−0.022 (−0.935)	−0.030 (−0.811)
Education	−0.003 (−1.126)	0.000 (0.164)
Marital status	0.039 (1.605)	0.070** (2.577)
Family members	0.031** (6.344)	0.029** (3.876)
Religion	0.071** (3.202)	0.112** (3.584)
Social insurance	0.060 (1.825)	0.031 (0.763)
Constant value	0.641** (11.407)	0.401** (4.931)
*R* ^2^	0.035	0.029
*F* –value	*F* (11, 2,897) = 10.507**	*F* (11, 2,440) = 6.780**
Sample size	2,909	2,452

* *p < 0.05*

** *p < 0.01*.

First, physical health status will have a positive impact on the willingness of urban respondents to have more children, but it will not affect the willingness of rural respondents to have more children. This could be because urban residents understand the “eugenics” policy better than rural residents. They are more concerned with the impact of genetic factors on their children's physical and mental health.

Second, the quality of marital status will have a positive impact on the willingness of urban residents to have multiple children, but this variable does not affect rural residents. The reason may be that urban residents have a more comprehensive understanding of marriage law, inheritance law, and other laws. They pay more attention to protecting their rights and interests by the law; thus, the better the marital status, the more willing they are to have a child. However, rural residents may not pay so much attention to the protection of the law due to their limited educational level; thereby, their willingness to bear children is not affected by marital status.

## Conclusion and Suggestion

This study examines whether family medical expenditures influence Chinese residents' willingness to have multiple children using a discrete choice model and regression analysis on data from the 2017 China General Social Survey (CGSS). According to the findings, the lower the level of medical expenditure, the lower the financial burden, and the greater the willingness to have more children. Furthermore, we found that residents' willingness to have multiple children is significantly influenced by their age. Residents between the ages of 40 and 49 have a considerably higher desire to have multiple children than those under 40, and healthier urban residents are more willing to have children. The health status of rural residents will not affect the willingness to have more children. The level of education is negatively correlated with the willingness to have more children. People with higher educational backgrounds are less likely to have more children. The willingness of urban residents to have more children is influenced by their marital status, whereas rural residents are unaffected. The prevalence of family cohabitation and religious beliefs encourage urban and rural residents to have multiple children. Ethnic minority residents are more willing to have children than Han residents, and rural residents are more inclined to have multiple children. This study draws the following enlightenment and recommendations based on the above conclusions.

At first, three perspectives can be developed by increasing medical security and reducing family medical expenditures. The first is to improve the convenience of medical treatment, improving the environment and accessibility of medical treatment and increasing the fairness of health services; the second is to control the price of medical services, effectively control or reduce residents' medical expenses, ensuring that residents are fair to enjoy basic medical and health services; third is to improve the level of medical security. It actively promotes social and medical insurance benefits and encourages everyone to participate. Conversely, it can organically integrate commercial medical insurance and social and medical insurance to achieve complementary effects.

Second, it enacted an additional policy to encourage residents to have three children, and it provided some incentives and subsidies to help them.

Some countries set up fertility funds to encourage childbirth, offering families “infant bonuses” and tax breaks (20). China can also implement tax incentive policies that lower taxes for families with two or three children, refund taxes and fees, and raise the personal income tax threshold. Furthermore, the government and work units provide subsidies to childbearing age groups, such as increasing housing provident funds and maternity allowances, including paying maternity insurance for employees. These incentives can improve their economic conditions and living standards, making them more willing to have two or three children.

Finally, residents should be introduced to and learn about population resources and fertility concepts through online social platforms and offline residences. The committees and units actively publicize China's current severe population aging, low fertility rate, and labor shortage, effectively promoting China's latest fertility policies and encouraging fertility to deviate from the traditional “fewer births and better births.” A more optimistic view of fertility.

## Data Availability Statement

Publicly available datasets were analyzed in this study. This data can be found here: Chinese General Social Survey (2017) http://cnsda.ruc.edu.cn/index.php?r=projects/view&id=93281139.

## Author Contributions

All authors listed have made a substantial, direct, and intellectual contribution to the work and approved it for publication.

## Conflict of Interest

The authors declare that the research was conducted in the absence of any commercial or financial relationships that could be construed as a potential conflict of interest.

## Publisher's Note

All claims expressed in this article are solely those of the authors and do not necessarily represent those of their affiliated organizations, or those of the publisher, the editors and the reviewers. Any product that may be evaluated in this article, or claim that may be made by its manufacturer, is not guaranteed or endorsed by the publisher.

## Footnotes

^1^“Single two–child” policy means the couples could have two children if one of them is an only child.
